# Single-Port One Anastomosis Sleeve Gastrectomy with Transit Bipartition: Initial Experience and Technique

**DOI:** 10.1007/s11695-024-07295-1

**Published:** 2024-05-21

**Authors:** Jason Widjaja, Jianjun Yang, Wenpei Dong, Rui Wang, Dongchao Yang, Zhicheng Song, Yan Gu

**Affiliations:** Department of General Surgery, Fudan University Affiliated Huadong Hospital, Shanghai, 200040 People’s Republic of China

**Keywords:** Sleeve gastrectomy, Single port, Weight loss, Bariatric surgery, Complications

## Abstract

**Background:**

Sleeve gastrectomy with transit bipartition (SG-TB) procedure has been gaining traction recently. While being a relatively novel procedure, it shows potentials to improve the standalone SG outcomes, such as diabetes remission and reflux. This article aims to show insights on performing SG-TB in one anastomosis fashion (SG-OATB) and single-port approach.

**Methods:**

Three patients who underwent laparoscopic single-port SG-OATB at our hospital were included. The parameters included in this study comprised of age, gender, height, weight, body mass index (BMI), type 2 diabetes mellitus (T2DM) assessment, gastroesophageal reflux disease (GERD) assessment, length of the small bowel, the duration of the procedure, and 30-day readmission rate.

**Results:**

The mean preoperative assessments for the three patients were as follows: two females vs. one male; age 38.7 ± 5.5 years old; weight 105.7 ± 5.4 kg; height 1.64 ± 0.11 m; BMI 39.3 ± 4.7 kg/m^2^; fasting blood glucose 6.7 ± 1.2 mmol/L; glycosylated hemoglobin level 7.1 ± 1.3%; GERD-Questionnaire score 6.3 ± 1.5; two patients with esophagitis grade A and B following endoscopy. The total duration of the procedure was 170.0 ± 26.5 min; there was no need for conversion to multiple-port in all patients. The 30-day readmission rate for all patients was 0%.

**Conclusion:**

In our small cases of patients, single-port SG-OATB is feasible and safe. We found the closure of the anastomosis defect to be most technically demanding. To understand better the outcome of single-port SG-OATB, studies with larger sample and longer follow-up will be needed in the future.

**Supplementary Information:**

The online version contains supplementary material available at 10.1007/s11695-024-07295-1.

## Introduction

Sleeve gastrectomy with transit bipartition (SG-TB) has been gaining attraction from many surgeons [1–5]. SG-TB is a relatively new surgical concept developed by Sergio Santoro and has been reported to have significant outcomes in terms of weight reduction, improvement of metabolic syndromes, and improvement of gastroesophageal reflux disease (GERD) [1–3]. The dual delivery system of the SG-TB carries a prospect of reducing nutritional complications and preserving overall clinical safety while maintaining excellent bariatric and metabolic outcome [1–3].

“Single anastomosis” or “one anastomosis” bariatric procedure is a popular technique due to its reduced difficulties when compared to the traditional Roux-en-Y technique. More to this, the lack of obvious mesenteric defect in one anastomosis procedure might reduce postoperative gastrointestinal complications due to adhesion and herniation [6].

**Fig. 1:**
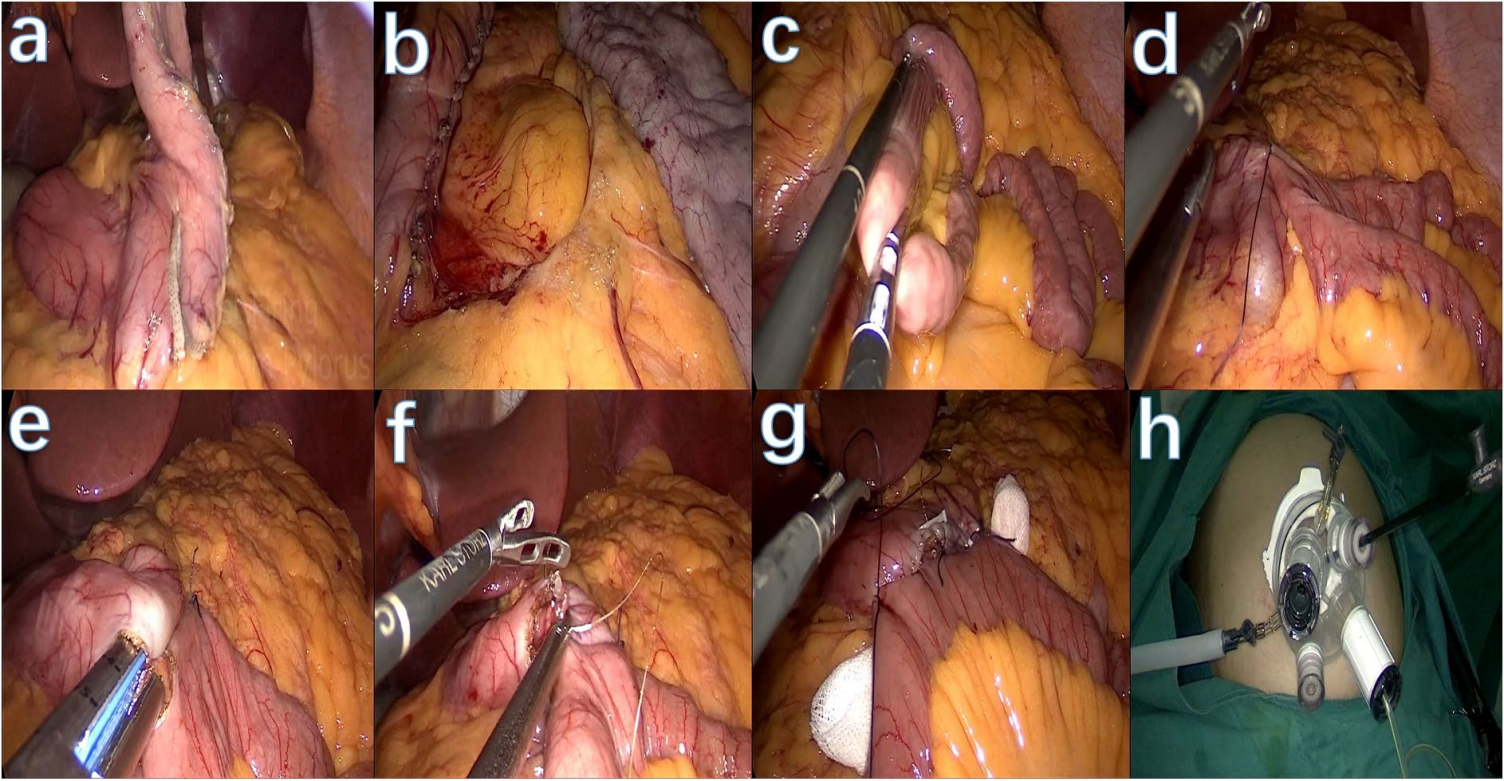
Graphics illustration of the single-port one anastomosis sleeve gastrectomy with transit bipartition. **a** Sleeve gastrectomy was performed starting from 6 cm away from the pylorus. **b** The entire sleeve was reinforced through full-layer suture. **c** The entire small bowel was measured and the anastomosis location is decided by maintaining 55–60% of the small bowel for the common limb. **d** The small bowel is fixed to the gastric body for easier anastomosis process. **e** The anastomosis is completed and the width is maintained at approximately 3 cm. **f** Closure of the anastomosis defect through sutures. **g** The gastrointestinal anastomosis is fixed at the upper- and lower-end. **h** The Quadri-port single-port trocar used

Single-port technique in bariatric surgery has been described widely, however not in the SG-TB procedure [7]. In this study, we share our initial experience in performing single-port one anastomosis SG-TB (SG-OATB) (Fig. 1).

## Methods

This study was approved by the Ethics Committee and institutional review of the Huadong Hospital Affiliated to Fudan University (2023K006) and was compliant with the Helsinki Declaration. All patients underwent detailed discussions regarding the pros and cons of laparoscopic single-port SG-OATB, and that conversion to multiple-port will be performed whenever deemed necessary. All patients signed the informed consent before the procedure.

None of the three patients in this study has received previous bariatric or abdominal surgeries (except for C-section), nor being debilitated with other diseases (such as cardiovascular, neurological, or malignancy diseases).

Our main criteria for SG-TB recommendation are mainly either type 2 diabetes mellitus (T2DM), GERD regardless of grading, history of constipation, or family history of gastric tumor. Furthermore, those who wish for pregnancy in the future will not be recommended for SG-TB procedure.

We routinely communicate in detail the pros and cons of the three procedures we perform, that is, standalone SG, gastric bypass, and SG-TB (DS mainly for conversion). Following our recommendations, each patient is free to choose the procedure they preferred (Table 1).

**Table 1:** Basic patients’ preoperative and intraoperative data

	Patient 1	Patient 2	Patient 3	Overall
Gender	Female	Female	Male	-
Age, y	33	39	44	38.7 ± 5.5
Weight, kg	92	90	135	105.7 ± 5.4
Height, m	1.58	1.57	1.76	1.64 ± 0.11
BMI, kg/m^2^	36.8	36.5	44.7	39.3 ± 4.7
T2DM assessment				
FBG, mmol/L	7.2	5.3	7.6	6.7 ± 1.2
HbA1C, %	7.4	5.7	8.2	7.1 ± 1.3
GERD assessment				
GERD-Q	6	8	5	6.3 ± 1.5
Endoscopy	Esophagitis A	Hiatal hernia Esophagitis B	Atrophic gastritis (Kimura-Takemoto C1)	
Total bowel length, cm	720	730	750	733.3 ± 15.3
Location for anastomosis, cm (measured from the Treitz ligament)	300	300	400	333.3 ± 57.7
Duration of the procedure, min	150	200	160	170.0 ± 26.5
30-day readmission	-	-	-	0

Data are shown as mean ± S.D. *BMI*, body mass index; *FBG*, fasting blood glucose; *HbA1c*, glycosylated hemoglobin; *GERD*, gastroesophageal reflux disease; *GERD-Q*, GERD-Questionnaire

### Data Selection

The preoperative and intraoperative parameters included in this study comprised of age, gender, height, weight, body mass index (BMI), type 2 diabetes mellitus (T2DM) assessment, gastroesophageal reflux disease (GERD) assessment, length of the small bowel, the duration of the procedure, and 30-day readmission rate.

### Single-Port OATB Procedure

Our single-port SG procedure with routine gastric suspension has been described with detail previously [8]. A Quadri-port LAGIPORT® SILS by LAGIS® (TRL-0220R) was used for our single-port procedure. For the SG-OATB procedure, the sleeve resection started 6 cm away from the pylorus and completed at around 1.5 cm away from the angle of His via a 36-Fr bougie (Covidien Endo GIA™ UltraXL linear stapler). The staple line reinforcement was completed through full-layer suturing (Ethicon 3–0 Stratafix™).

For the OATB part, we measured the total length of the small bowel and maintained the common limb at 55–60% of the total small bowel (for patients without diabetes as well as controlled diabetes). The gastrointestinal anastomosis started approximately 3 cm away from the pylorus. The gastrointestinal anastomosis was approximately 2.5 cm in width and the defect was closed with sutures. A brief video of our single-port SG-OATB procedure is available (Video).

The size of the single-port incision was 2.5–3 cm approximately. Simple interrupted sutures using 3–0 sutures were performed to close the incision.

## Results

This study includes three patients (two female and one male) that underwent single-port SG-OATB in our hospital. The mean preoperative data are as follows: age 38.7 ± 5.5 years, weight 105.7 ± 5.4 kg, height 1.64 ± 0.11 m, BMI 39.3 ± 4.7 kg/m^2^, fasting blood glucose (FBG) 6.7 ± 1.2 mmol/L, HbA1c 7.1 ± 1.3%, and GERD-Questionnaire (GERD-Q) 6.3 ± 1.5. With regard to the preoperative endoscopic results, one patient was with esophagitis grade A, one patient with esophagitis grade B and hiatal hernia, and one patient with atrophic gastritis (Kimura-Takemoto C1).

Intraoperatively, the total small bowel length vs. the location of the anastomosis (measured from the Treitz ligament) for the three patients were as follows: 720 vs. 300 cm, 730 vs. 300 cm, and 750 vs. 400 cm, respectively. The total duration of the procedure for the three patients was as follows: 150 min, 200 min (with hiatal hernia repair), and 160 min, respectively.

No patients needed conversion to multiple-port in this study. Abdominal drainage was placed in all patients. All patients were discharged at postoperative day 4. There were no patients readmitted to any hospital 30 days postoperatively.

## Discussion

To our knowledge, this is the first study to report the technical feasibility of single-port OATB. Before performing the single-port SG-OATB, we have experienced over 400 cases of single-port SG. Our initial reservation with single-port SG-OATB was with the measurements of the small bowel as this maneuver will be restricted by the angulation from the single-port. Interestingly, the measurement of the small bowel was performed and completed effortlessly. The stapling of the gastrointestinal anastomosis was also completed without much difficulties. To our surprise, the most difficulties we experienced were during the closure of the anastomosis defect, especially as we want to confirm that the mucosa of the stomach and the small bowel was sutured. The difficulty in closing the anastomosis defect could be due to the size of the defect; as the defect becomes smaller, the angulation for the instrument maneuvers becomes smaller as well.

For patient 1, she had a FBG of 7.2 mmol/L and HbA1c level of 7.4% without medications (recently diagnosed T2DM), esophagitis grade A, and history of constipation, while for patient 2, she was without diabetes, however with high GERD-Q score and esophagitis grade B, as well as history of constipation and with family history of gastric cancer. For these two patients, the SG-OATB procedure was performed mainly to reduce the pressure of the sleeve; thus, the gastrointestinal anastomosis was located 300 cm away from the Treitz ligament maintaining approximately 60% of the common limb. Although more studies are needed to better support the anti-GERD outcome of TB, several studies have reported that TB might have beneficial anti-GERD effect [9, 10]. For patient 3, he had uncontrolled HbA1c and FBG level under one medication; thus, the gastrointestinal anastomosis was located further away from the Treitz ligament (400 cm away from the Treitz ligament).

This study, being a brief report to provide some insights with regard to single-port approach on SG-OATB, has several limitations. The short follow-up made us unable to report the outcome of the procedure. However, none of the three patients has 30-day readmission. Furthermore, SG-TB is yet a standardized bariatric procedure; thus, it will be difficult for standardization. Longer follow-up with larger sample will be needed in the future.

Single-port procedure has several benefits when compared to the multiple-port technique. Most importantly, single-port technique showed non-inferiority when compared to multiple-port [11]. Most obviously, the cosmetic aspect will be improved by single-port approach. However, the risk for incisional hernia might be high in single-port technique if patients’ selection is not rigorous. Patients with subcutaneous abdominal fat thickness of 5–6 cm or more should not be recommended for single-port approach. With adequate suture technique, the issue of incisional hernia can be addressed sufficiently. Indeed, a study by Lee et al. reported that the single-port approach did not increase the rate of incisional hernia when compared to the multiple-port [12].

## Conclusion

To our knowledge, we describe the feasibility of the single-port SG-OATB for the first time. Further evaluation remains needed with greater number of cases and longer follow-up. We would like to further note that single-port technique is an option for bariatric surgery; however, it is not the goal. Therefore, we make certain to all patients that conversion to multiple-port will be performed when deemed necessary.

### Supplementary Information

Below is the link to the electronic supplementary material.

Supplementary file1 (MP4 322332 kb)

## Data Availability

The datasets generated during and/or analysed during the current study are available from the corresponding author on request.
